# Prenatal Exposure to Delta-9-tetrahydrocannabinol (THC) Alters the Expression of miR-122-5p and Its Target *Igf1r* in the Adult Rat Ovary

**DOI:** 10.3390/ijms23148000

**Published:** 2022-07-20

**Authors:** Annia A. Martínez-Peña, Kendrick Lee, Madison Pereira, Ahmed Ayyash, James J. Petrik, Daniel B. Hardy, Alison C. Holloway

**Affiliations:** 1 Department of Obstetrics and Gynecology, McMaster University, Hamilton, ON L8S 4K1, Canada; martia77@mcmaster.ca (A.A.M.-P.); ayyasha@mcmaster.ca (A.A.); 2 The Children’s Health Research Institute, The Lawson Health Research Institute, Departments of Obstetrics and Gynecology and Physiology and Pharmacology, Western University, London, ON N6A 3K7, Canada; klee843@uwo.ca (K.L.); daniel.hardy@schulich.uwo.ca (D.B.H.); 3 Department of Biomedical Sciences, University of Guelph, Guelph, ON N1G 2W1, Canada; mperei02@uoguelph.ca (M.P.); jpetrik@uoguelph.ca (J.J.P.)

**Keywords:** cannabis, developmental toxicology, delta-9-tetrahydrocannabinol, ovarian function, miR-122-5p, insulin-like growth factor 1 receptor

## Abstract

As cannabis use during pregnancy increases, it is important to understand its effects on the developing fetus. Particularly, the long-term effects of its psychoactive component, delta-9-tetrahydrocannabinol (THC), on the offspring’s reproductive health are not fully understood. This study examined the impact of gestational THC exposure on the miRNA profile in adult rat ovaries and the possible consequences on ovarian health. Prenatal THC exposure resulted in the differential expression of 12 out of 420 evaluated miRNAs. From the differentially expressed miRNAs, miR-122-5p, which is highly conserved among species, was the only upregulated target and had the greatest fold change. The upregulation of miR-122-5p and the downregulation of its target insulin-like growth factor 1 receptor (*Igf1r*) were confirmed by RT-qPCR. Prenatally THC-exposed ovaries had decreased IGF-1R-positive follicular cells and increased follicular apoptosis. Furthermore, THC decreased *Igf1r* expression in ovarian explants and granulosa cells after 48 h. As decreased IGF-1R has been associated with diminished ovarian health and fertility, we propose that these THC-induced changes may partially explain the altered ovarian follicle dynamics observed in THC-exposed offspring. Taken together, our data suggests that prenatal THC exposure may impact key pathways in the developing ovary, which could lead to subfertility or premature reproductive senescence.

## 1. Introduction

The use of cannabis, which is the generic term that denotes several preparations of the plant *Cannabis sativa,* has increased considerably in recent decades [1]. The increase in cannabis use has been more prominent in developed countries, where there is a growing tendency towards legalization, and specifically among the younger population, whose perception of cannabis as a harmful drug has decreased over the past decade [1]. Regarding cannabis use during pregnancy, several studies have confirmed that consumption during gestation has also increased during the last decades [2,3,4]. Ko and colleagues reported that 70% of pregnant and nonpregnant women in the US believe that there is little to no harm in using cannabis once or twice per week [5], and a longitudinal prospective study discovered that 48% of women in the UK who used cannabis in the year prior to their pregnancy continued to smoke throughout gestation [6]. Despite advice against its use during pregnancy [7], cannabis is often used to mitigate pregnancy-related symptoms such as pain, nausea and vomiting [8,9,10]. Studies that have investigated the prevalence of cannabis use in developed countries revealed that approximately 4–7% of women reported using cannabis during pregnancy [3,4,11,12]. However, given that most studies rely solely on self-reporting, it is likely that these data underestimate the actual prevalence of cannabis use during gestation [13,14].

Many researchers have demonstrated that prenatal cannabis use is associated with an increased risk of stillbirth [15,16], preterm birth [4,15,17,18], being small for gestational age [17,18,19,20], low birthweight [17,21,22,23], increased admission to neonatal intensive care units [18,20] and death within one year of birth [17]. In addition, prenatal exposure to cannabis may also result in long-term alterations in the offspring’s health [24]. Considering that delta-9-tetrahydrocannabinol (THC), the psychoactive component of cannabis [25], efficiently crosses the placental barrier and can be detected in cord blood and fetal tissue [26], this compound could have direct effects on the developing organs, which are particularly sensitive to exogenous chemicals [27,28]. It is known that adverse prenatal conditions are associated with an increased risk of disease later in life, and it has been proposed that while the fetus adapts to these adverse conditions by favoring the development of organs that ensure survival, this reprogramming has harmful long-term effects on other organs, including the ovaries [29]. Indeed, it has been shown that prenatal conditions such as maternal undernutrition, hormonal imbalance or exposure to exogenous chemicals have the potential to alter ovarian development, with major implications for reproductive health and fertility later in life [30].

Studies have suggested that prenatal cannabinoid exposure results in long-lasting neurobehavioral, metabolic, cardiovascular and reproductive abnormalities in offspring [31,32,33,34,35,36,37,38]. Given that these effects are observed long after exposure, one of the proposed mechanisms is that cannabis may cause epigenetic changes in the developing fetus [24]. Alterations in epigenetic regulation can be a consequence of exposomes, including environmental factors (e.g., nutrition and oxygen levels) and/or exogenous compounds (e.g., drugs), which results in heritable phenotypic changes without affecting the DNA sequence [39]. Indeed, several studies have shown that cannabis constituents (i.e., THC and cannabidiol) have the ability to induce epigenetic changes such as altered DNA methylation, histone modifications and microRNA (miRNA) expression in multiple tissues, resulting in long-lasting effects [40,41,42,43,44,45,46,47,48].

MiRNAs are small, endogenous, noncoding single-stranded RNA molecules with a length of approximately 22 to 24 nucleotides, which act as posttranscriptional regulators of gene expression [49,50]. Mature miRNAs can regulate gene expression through different mechanisms, including mRNA cleavage and translational inactivation [51]. Given that miRNAs can bind to the 3′-UTR region with partial sequence homology, a single miRNA may have multiple mRNA targets, and a single mRNA transcript may be targeted by several different miRNAs [52]. MiRNAs are involved in the regulation of several cellular processes, including differentiation, proliferation, apoptosis and hormone biosynthesis and secretion [53,54]. In the ovary, miRNAs play an important role in steroidogenesis, oocyte maturation, ovulation, luteinization, follicular development and atresia, and their dysregulation has been associated with disorders such as polycystic ovary syndrome (PCOS), premature ovarian failure (POF) and ovarian cancer [55,56].

A previous study from our research group revealed that prenatal exposure of rats to THC resulted in altered ovarian follicle dynamics and vascularization in the adult offspring [57]. Considering that miRNAs are involved in the regulation of key ovarian processes, and that cannabis constituents have been shown to affect miRNA expression, the aims of this study were to assess the effect of prenatal exposure to THC on the ovarian miRNA profile and to determine the possible impact of these changes on ovarian health and function.

## 2. Results

### 2.1. Gestational Exposure to THC Alters miRNA Profile in Adult Rat Ovaries

**Figure 1:**
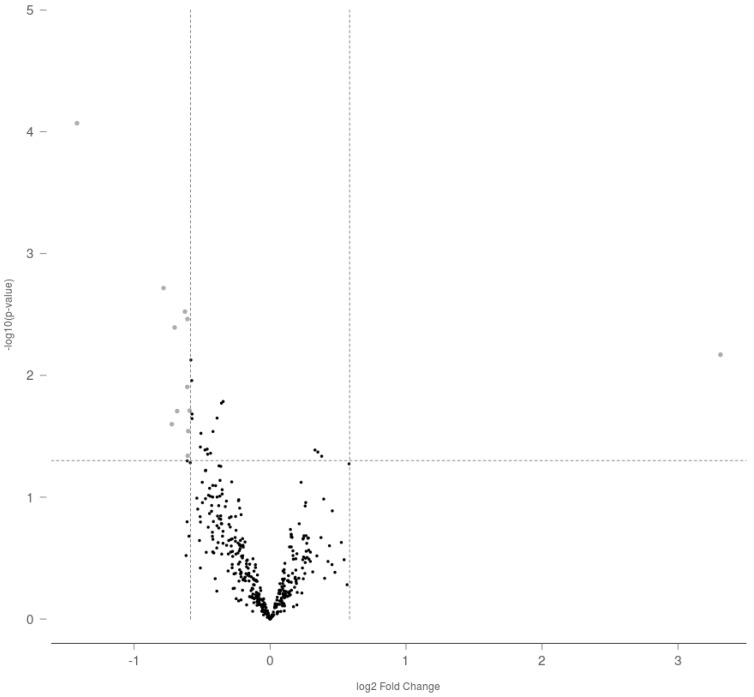
Volcano plot representation of differential miRNA expression as a result of gestational exposure to THC in adult rat ovaries. The dotted lines represent the fold change (≥1.5 or ≤−1.5) and *p*-value (<0.05) cutoff values selected to determine statistical difference.

The differential expression of miRNA targets in adult rat ovaries as a result of gestational exposure to THC is shown in Figure 1. Of the 420 miRNAs evaluated by the Nanostring assay, prenatal exposure to THC altered the expression of 12 miRNA targets (fold change ≥ 1.5, *p* < 0.05).

From the 12 differentially expressed miRNAs, 11 were downregulated (miR-154-5p, miR-214-5p, miR-3552, miR-18a-5p, miR-296-3p, miR-874-3p, miR-211-5p, miR-20b-3p, miR-598-3p, miR-330-5p and miR-190b-5p) and only miR-122-5p was upregulated (Table 1). For this reason, along with the fact that it exhibited the greatest fold change in expression, miR-122-5p (miR-122) was chosen for further evaluation.

### 2.2. Prenatal THC-Exposure Increases Ovarian miR-122-5p Expression in Adult Offspring

**Figure 2:**
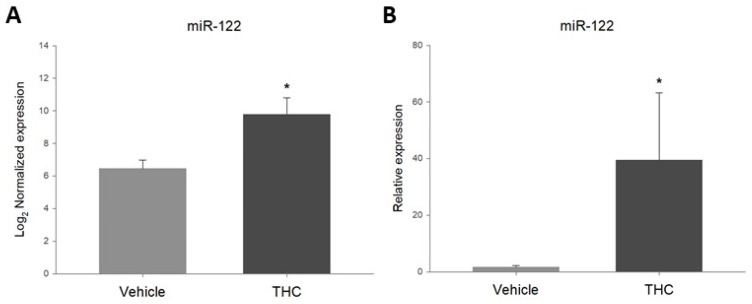
Differential expression of miR-122-5p (miR-122) in adult rat ovaries prenatally exposed to THC analyzed with (**A**): Rat v1.5 miRNA assay (Nanostring Technologies, Seattle, DC, USA), nSolver^®^ and ROSALIND^®^ analysis software, and (**B**): miRCURY™ LNA miRNA PCR assay system (Qiagen, Hilden, Germany) and SigmaPlot^®^ (mean + SEM; *N* = 5, * *p* < 0.05).

In order to confirm the increase in miR-122 in the prenatally THC-exposed ovaries, the expression of this target was assessed by RT-qPCR. As shown in Figure 2B, RT-qPCR results revealed that miR-122 was significantly upregulated in the ovaries from THC-exposed offspring, similar to the results obtained with the Nanostring assay (Figure 2A).

### 2.3. Gestational Exposure to THC Decreases Igf1r Expression in the Adult Ovary

**Figure 3:**
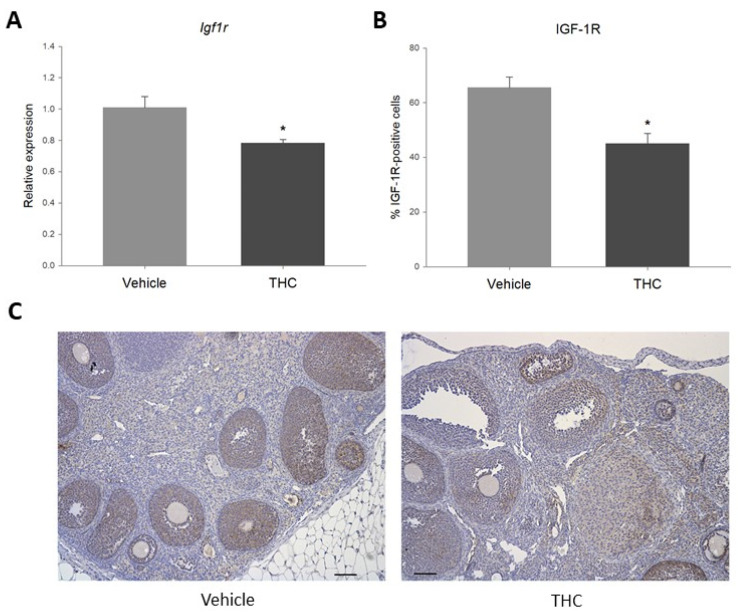
(**A**). Relative expression of *Igf1r* in prenatally THC-exposed adult rat ovaries. (**B**). Percentage of IGF-1R-positive ovarian follicular cells in prenatally THC-exposed adult rat ovaries. (**C**). IGF-1R protein levels in prenatally THC-exposed adult rat ovaries (mean + SEM; *N* = 5, * *p* < 0.05).

Changes in miRNA expression may affect the expression of their target genes [51]. Given that gestational THC-exposure resulted in a significant increase in miR-122, the online databases TargetScan and miRTarBase were used to determine validated miR-122 target genes involved in the regulation of ovarian processes. Insulin-like growth factor 1 receptor (*Igf1r*) was identified as a key ovarian miR-122 target based on its role in steroidogenesis, folliculogenesis, angiogenesis, cell proliferation and apoptosis [58,59]. Real-time qPCR revealed that prenatal exposure to THC significantly decreased the relative expression of *Igf1r* in the ovary, as well as the percentage of IGF-1R-positive follicular cells, as determined by immunohistochemistry (IHC) (Figure 3).

### 2.4. Prenatal Exposure to THC Increases Follicular Apoptosis in the Adult Ovary

**Figure 4:**
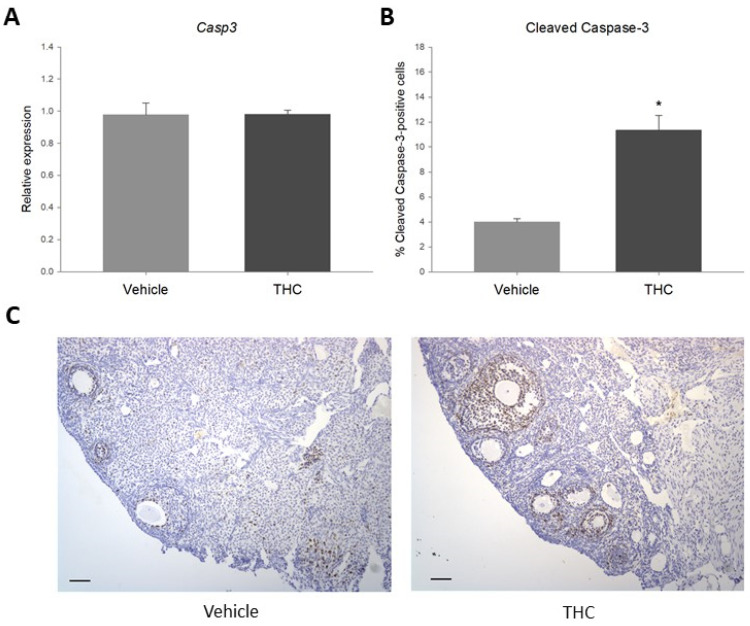
(**A**). Relative expression of *Casp3* in prenatally THC-exposed adult rat ovaries. (**B**). Percentage of cleaved caspase-3-positive ovarian follicular cells in prenatally THC-exposed adult rat ovaries. (**C**). Cleaved caspase-3 protein levels in prenatally THC-exposed adult rat ovaries (mean + SEM; *N* = 5, * *p* < 0.05).

It has previously been established that IGF-1/IGF-1R signaling plays an essential role in follicular growth and atresia, mostly by regulating granulosa cell proliferation and apoptosis [58]. Considering that gestational exposure to THC resulted in a significant decrease in IGF-1R, the expression of the apoptotic marker caspase-3 was evaluated. As shown in Figure 4, while there were no changes in the steady-state mRNA expression of *Casp3*, gestational exposure to THC significantly increased the percentage of cleaved caspase-3-positive ovarian follicular cells.

### 2.5. Acute Exposure to THC Decreases Expression of Igf1r in the Ovary

**Figure 5:**
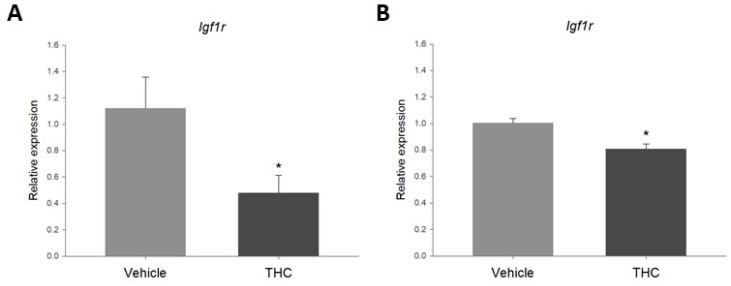
(**A**). Relative expression of *Igf1r* in rat ovary explants exposed to 3 μM THC for 48 h. (**B**). Relative expression of *Igf1r* in spontaneously immortalized rat granulosa cells exposed to 3 μM THC for 48 h (mean + SEM; *N* = 5, * *p* < 0.05).

In order to determine if THC had a direct effect on the expression of *Igf1r* in the ovaries, ex vivo and in vitro models were used. While it is possible to observe short-term effects of direct THC exposure on gene expression in both models, the use of ovarian explants represents a microenvironment and cell diversity that more closely resemble in vivo conditions. On the other hand, the use of an immortalized cell line provides information on the effect of the compound on that specific cell type. Ovarian explants and spontaneously immortalized rat granulosa cells were cultured and exposed to 3 μM THC for 48 h. As observed in Figure 5, exposure to 3 μM THC significantly decreased the relative expression of *Igf1r* in the ovary explants (Figure 5A) and the granulosa cells (Figure 5B).

## 3. Discussion

As the use of cannabis during pregnancy increases, it is important to understand the possible effects of its constituents on the developing fetus. The present study examined the effect of gestational exposure to THC on the miRNA profile in adult rat ovaries, as a proposed mechanism through which THC-induced changes in miRNA expression could influence the altered ovarian follicle dynamics and vascularization previously observed in these offspring [57]. Prenatal exposure to THC resulted in the differential expression of 12 miRNA targets, as determined by a Nanostring assay that evaluated the expression of 420 biologically relevant rat miRNAs. While there are several studies that report in vivo alterations of miRNA expression as a consequence of direct exposure of primates and rodents to THC [60,61,62], work from our group has also shown that gestational exposure to THC resulted in altered miRNA profiles in the liver of adult rat offspring [37]. Similarly, in this study we report an altered miRNA profile in the ovaries of THC-exposed female offspring.

Of the 420 miRNAs included in the Nanostring assay, miR-122-5p (miR-122) was the only upregulated target, as well as having the largest fold-change compared to the controls. As miR-122 is a highly conserved miRNA among multiple species, including rat and human [63], this target was chosen for further evaluation. RT-qPCR confirmed the upregulation of miR-122 in the prenatally THC-exposed ovaries. There is growing evidence that suggests miR-122 is involved in the regulation of cell proliferation, differentiation, migration and apoptosis [64,65]. A recent study revealed increased expression of miR-122 in atrophic chicken ovaries compared to healthy ones [63]. Flow cytometry revealed that granulosa cell apoptosis was significantly decreased by a miR-122 inhibitor, while treatment with a miR-122 mimic increased apoptosis and caspase-3 protein levels in vitro [63]. Similarly, Zhang and colleagues recently reported increased granulosa cell apoptosis in a rodent model of primary ovarian insufficiency following treatment with a miR-122 mimic, while a miR-122 inhibitor reduced granulosa cell apoptosis [66]. Furthermore, Menon and colleagues observed that miR-122 plays an important role in the regulation of the luteinizing hormone receptor mRNA binding protein (LRBP) during FSH-induced follicular growth, and therefore may impact folliculogenesis and ovulation [67,68].

To evaluate the effects of the upregulation of miR-122 in the ovaries, the online databases TargetScan and miRTarBase were used to identify validated miR-122 targets involved in the regulation of ovarian processes. The validated miR-122 target insulin-like growth factor 1 receptor (*Igf1r*) was selected for further evaluation. Studies have reported that miR-122 can directly bind to and decrease *Igf1r* transcript in vitro [69,70,71]. Moreover, IGF-1R has been shown to play a role in the regulation of folliculogenesis, ovulation, angiogenesis and granulosa cell proliferation and apoptosis [58,72]. IGF-1R is a receptor tyrosine kinase that, among other functions, activates the AKT pathway, linked to prevention of apoptosis, and the ERK pathway, which is associated with growth and proliferation [73]. IGF-1R expression has been detected in most structures of the rodent ovary, including the stroma, oocytes, corpus luteum and theca cells, with the strongest staining found in granulosa cells [58]. Gestational exposure to THC significantly decreased mRNA expression of *Igf1r* and the percentage of IGF-1R-positive follicular cells in the ovaries. These results are in accordance with the observed increase in miR-122 and with studies that have reported the downregulation of *Igf1r* as a result of miR-122 overexpression [69,70,71]. Furthermore, the decrease in *Igf1r* expression appears to be directly attributable to the effect of THC on the ovary, as this compound significantly decreased the relative expression of *Igf1r* in ovary explants and spontaneously immortalized rat granulosa cells (SIGCs) exposed to 3 μM for 48 h. Considering the key role of IGF-1R in the ovary [58,59], it is possible that a similar mechanism may partially explain the adverse reproductive outcomes such as an increased risk of infertility due to ovulatory abnormalities [74], fewer and poorer quality oocytes and lower pregnancy rates by in vitro fertilization (IVF) [75] that have been reported in adult cannabis users.

Protein levels of IGF-1R have been shown to be reduced in granulosa cells from patients with PCOS and ovarian tissue of PCOS rat models [76]. This reduction was linked to decreased AKT phosphorylation and increased caspase-3 activity, suggesting that increased granulosa cell apoptosis plays a role in the abnormal folliculogenesis and anovulation in PCOS [76]. It has also been shown that knockdown of *Igf1r* in granulosa cells results in a lack of response to FSH in vitro, and that inhibiting IGF-1R activity in vivo prevents eCG-induced follicular growth [72]. Moreover, Baumgarten and colleagues demonstrated that female mice with a conditional *Igf1r* knockdown in granulosa cells were sterile, with small ovaries lacking antral follicles, even after stimulation with gonadotropin [58]. Accordingly, these animals failed to ovulate after a superovulation protocol, and their granulosa cells expressed significantly lower levels of preovulatory markers [58]. Similar to what has been observed in PCOS models, ovaries with reduced IGF-1R had impaired AKT activation, as well as increased levels of caspase-3-dependent apoptosis in follicles transitioning from the primary to the large secondary stages [58]. The authors concluded that the lack of IGF-1R signaling in the granulosa cells resulted in increased apoptosis and failure to respond to FSH, which in turn led to the complete arrest of folliculogenesis and the subsequent loss of fertility. In the present study, in addition to reduced *Igf1r* expression, THC-exposed offspring had an increased percentage of cleaved caspase-3-positive follicular cells in the ovaries, suggesting an increase in apoptosis. In addition, IHC revealed that cleaved caspase-3 was predominantly expressed in granulosa cells, which could suggest increased granulosa cell apoptosis, an important factor of follicular atresia [77].

The reduction in *Igf1r* expression may also help explain the altered follicle dynamics in the ovaries of THC exposed offspring previously reported by our group [57]. In the same cohort of animals, we observed that ovaries from THC-exposed offspring had a significant increase in transitioning follicles without any differences in later stages of follicular development, suggesting a portion of the follicles did not continue to develop. While this was partially attributed to the reduced blood vessel formation in these ovaries, it is possible that the increased apoptosis revealed in the present study also contributed to the impaired follicular development. The reduced vascularization of these ovaries was associated with a decrease in VEGF and VEGFR2 expression and an increase in TSP-1. This is interesting considering it has been shown that activation of IGF-1R results in increased VEGF expression and secretion in bovine luteal cells [78]. Additionally, it has been demonstrated that in vitro treatment of immortalized rat granulosa cells with TSP-1 increases the expression of pro-apoptotic factors [79], while VEGF-VEGFR2 signaling appears to have the opposite effect [80]. It was therefore proposed that the dysregulation of these factors could manifest as increased apoptosis (atresia) and ultimately a loss of developing follicles [57]. Although there were no statistically significant differences in the number of atretic follicles in the THC-exposed ovaries (*p* = 0.197), this could be a reflection of the small sample size. It is also possible that the increase in apoptosis may manifest more clearly as follicle loss as the animal ages. Indeed, a rodent model with granulosa cell-specific silencing of *Vegf* revealed a decrease in *Igf1r* expression and increased granulosa cell apoptosis [81]. According to the authors, these animals were subfertile, and the effect seemed to be greater as the mice aged [81]. It would therefore be of great interest to continue monitoring the ovarian follicle reserve as the animals approach reproductive senescence and to assess the THC-exposed offspring’s fertility, since the observed effects may have an impact on other reproductive aspects such as oocyte quality.

## 4. Materials and Methods

### 4.1. Animals

Pregnant Wistar rats were purchased from Charles River (La Salle, St. Constant, QC, Canada) and maintained at 22 °C at Western University Animal House Facility on a 12:12 h light:dark cycle with access to food and water ad libitum throughout the experiment. All animal experiments were done based on the approved animal use protocol by the subcommittee of the Canadian Council of Animal Care, Western University (AUP# 2019-126) in accordance with the ARRIVE guidelines (https://arriveguidelines.org, accessed on 16 July 2022). Dams were randomly assigned to receive a daily intraperitoneal (IP) injection of either vehicle (1:18 cremophor:saline) or 3 mg/kg THC (Sigma-Aldrich, St Louis, MO, USA) from gestation day (GD) 6 to GD22. This dose has been shown to result in maternal blood concentrations (8.6–12.4 ng/mL) comparable to those detected after moderate cannabis smoking in adults (13–63 ng/mL), as well as in aborted fetal tissue (4–287 ng/mL) after maternal cannabis use [82,83,84]. We have previously demonstrated that 3 mg/kg THC per day does not cause fetal demise, alterations in litter size or gestational length, or maternal weight gain [85]. Dams were allowed to deliver normally, and litters were randomly culled to four female pups and four male pups each. At 6 months of age, prenatal THC-exposed offspring were euthanized by IP pentobarbital overdose. Per female, one ovary was fixed in 10% formalin and embedded in paraffin for histological analysis, while the other ovary was flash frozen in liquid nitrogen to evaluate miRNA and mRNA expression.

### 4.2. Nanostring Analysis

To determine the effect of gestational THC exposure on the miRNA profile in adult rat ovaries, RNA was extracted from the ovaries of THC-exposed offspring with the use of a mirVana™ miRNA isolation kit (Thermo Fisher Scientific, Waltham, MA, USA). RNA quality was assessed using a 2100 Bioanalyzer (Agilent, Santa Clara, CA, USA), obtaining RNA integrity numbers that ranged from 7.4 to 9.3. The Rat v1.5 miRNA Assay (Nanostring Technologies, Seattle, WA, USA) was used to determine the expression levels of 420 biologically relevant rat miRNA targets according to the manufacturer’s instructions. Results were analyzed with the use of nSolver^®^ analysis software and ROSALIND^®^. A statistically significant effect was considered to be a fold change of ≥1.5 or ≤−1.5 and a *p* value < 0.05.

### 4.3. miRNA Real-Time Quantitative PCR

In order to confirm the results obtained with the Nanostring assay, the expression of the miRNA target with the greatest fold change (miR-122-5p) was evaluated by real-time quantitative PCR (RT-qPCR). For this, the miRCURY™ LNA miRNA PCR Assay system (Qiagen N.V., Hilden, Germany) was used. Complementary DNA (cDNA) was synthesized from the extracted RNA with the use of a miRCURY™ LNA RT kit (Qiagen). The cDNA was then amplified and detected with the use of a miRCURY™ LNA SYBR^®^ Green PCR kit (Qiagen) and a CFX384 Touch™ Real-Time PCR Detection System (Bio-Rad Laboratories, Hercules, CA, USA). RT-qPCR results were analyzed with the 2^−^^∆∆CT^ method [86] using RNU5G snRNA and miR-191-5p as internal references (Table 2). These reference genes were chosen based on previous studies and showed the lowest variation between vehicle and THC-exposed samples when analyzed using RefFinder [87,88,89].

### 4.4. Immunohistochemistry

Paraffin-embedded ovaries were sectioned (8 μm) and deparaffinized using reagent-grade xylene, and subjected to a series of decreasing ethanol concentrations for rehydration. Endogenous peroxidase activity was quenched through a 10-min incubation period in 3% hydrogen peroxide, followed by antigen retrieval using 10 mM sodium citrate buffer with Tween 20 (0.05%) for 12 min. To reduce nonspecific binding of antibodies, samples were blocked with 5% bovine serum albumin (with 0.02% sodium azide) for 10 min at room temperature. Sections were exposed to either rabbit polyclonal anti-IGF-1R (Abcam, Cambridge, UK; 1:400) or anti-cleaved caspase-3 (Cell Signaling Technology, Danvers, MA, USA; 1:300) overnight at 4 °C. Slides were then incubated with biotinylated anti-rabbit secondary antibody (Thermo Fisher Scientific; 1:100) for 2 h at room temperature followed by Extravidin (Sigma-Aldrich; 1:50) for 1 h at room temperature. Antigens were visualized using 3,3′-diaminobenzidine tetrahydrochloride (DAB; Sigma-Aldrich), and tissues were counterstained with hematoxylin. Slides were imaged using bright-field microscopy at 200x magnification. The percentage of immunopositive follicular cells was quantified by the same individual, who was blinded to the treatment group until all the data had been collected, with the use of an integrated morphometry software (MetaMorph Inc., Nashville, TN, USA). The average of 5 fields of view/ovary was used to calculate the percentage of immunopositive cells.

### 4.5. Ovarian Explant Culture

For ex vivo experiments, ovaries were collected from nulliparous Wistar rats (268.09 ± 2.96 g) and transferred to sterile D-PBS (Corning Inc., New York, NY, USA). In a biosafety cabinet, ovaries were trimmed of fat, washed with D-PBS and cut into four equal pieces using a sterile blade. Each piece was transferred to a single well in a 24-well plate containing DMEM/F12 media with L-glutamine (Corning) supplemented with 10% fetal bovine serum (FBS) and 2% penicillin/streptomycin (Thermo Fisher Scientific). After 24 h of culture, explants were exposed to vehicle or 3 μM THC for 48 h, changing the media daily. This concentration of THC was based on a pharmacokinetic study that reported similar levels in the serum of cannabis users [90].

### 4.6. Cell Culture

For in vitro experiments, spontaneously immortalized rat granulosa cells (SIGCs) were cultured in DMEM/F12 media with L-glutamine (Corning Inc.) supplemented with 10% fetal bovine serum (FBS) and 2% penicillin/streptomycin. As with ex vivo experiments, cells were cultured with either vehicle or 3 μM THC for 48 h after confirming this concentration had no effect on cell viability (data not shown).

### 4.7. RNA Isolation and RT-qPCR

To evaluate gene expression, total RNA was extracted from THC-exposed ovary explants and SIGCs. Briefly, samples were homogenized in TRIzol™ reagent (Thermo Fisher Scientific) by sonication, and RNA was extracted by precipitation with isopropanol and subsequent ethanol washes. RNA concentration and purity were assessed using a NanoDrop™ One micro-UV/vis spectrophotometer (Thermo Fisher Scientific), and cDNA was synthesized with the use of a high capacity cDNA reverse transcription kit (Thermo Fisher Scientific).

In order to confirm the effects of miRNA dysregulation, the online databases miRTarBase (https://mirtarbase.cuhk.edu.cn/~miRTarBase/miRTarBase_2022/php/index.php, accessed on 16 July 2022) and TargetScan (https://www.targetscan.org/vert_80/, accessed on 16 July 2022) were used to select validated target genes involved in essential ovarian processes. With this in mind, the expression of the validated miR-122-5p target insulin-like growth factor 1 receptor (*Igf1r*) was determined. In addition, given that both miR-122-5p and IGF-1R are involved in the regulation of apoptosis [58,66], and that alterations in follicle dynamics were previously observed in the prenatally THC-exposed ovaries [57], the expression of the apoptotic marker caspase-3 was evaluated.

For the ovaries from prenatally THC-exposed offspring, the ovary explants and the SIGCs, RT-qPCR was performed using PerfeCTa SYBR^®^ Green FastMix (Quantabio, Beverly, MA, USA) and the CFX384 Touch™ real-time PCR detection system (Bio-Rad). RT-qPCR results were analyzed with the 2^−∆∆CT^ method using beta-2-microglobulin (*B2m*) and hypoxanthine phosphoribosyltransferase 1 (*Hprt1*) as internal references (Table 3).

### 4.8. Statistical Analysis

For in vivo assessments, one female offspring per dam was included in the statistical analysis. For ex vivo and in vitro assessments, results from 5 independent experiments are presented. The Nanostring assay results were analyzed with the use of nSolver^®^ and ROSALIND^®^ analysis software, considering a fold change ≥ 1.5 and a *p*-value < 0.05 as statistically different. For miRNA and mRNA RT-qPCR, as well as for immunohistochemical evaluation, statistical analyses were performed using SigmaPlot^®^. A Student’s t-test was performed to determine statistically significant differences (*p* < 0.05) between vehicle- and THC-exposed samples.

## 5. Conclusions

We have previously shown that prenatal exposure to delta-9-tetrahydrocannabinol (THC), the psychoactive component of cannabis, resulted in altered follicle dynamics and vascularization in the adult rat ovary. In this study, we propose that gestational exposure to THC altered miRNA expression in the ovary, which in turn affected ovarian health in adulthood. Particularly, prenatal THC exposure resulted in a significant increase in the expression of miR-122-5p and a significant decrease in the expression of its validated target gene insulin-like growth factor 1 receptor (*Igf1r*). Importantly, decreased IGF-1R expression has been linked to increased apoptosis and abnormal folliculogenesis, both of which have been observed in the THC-exposed offspring. Taken together, these data suggest that prenatal THC-exposure may impact key pathways in the developing ovary that could lead to subfertility or premature reproductive senescence. As the use of cannabis during pregnancy increases, it is important to understand the safety of this drug and its constituents, as well as the possible long-term effects on the offspring’s endocrine and reproductive health.

## Figures and Tables

**Table 1:** List of differentially expressed miRNA targets in the adult rat ovary as a result of prenatal THC exposure.

Accession Number	Target	Effect	Fold Change	*p* Value
MIMAT0000827	rno-miR-122-5p	Upregulated	9.93741	0.00676
MIMAT0000856	rno-miR-154-5p	Downregulated	−2.67658	8.50 × 10^−5^
MIMAT0000885	rno-miR-214-3p	Downregulated	−1.72175	0.00192
MIMAT0017813	rno-miR-3552	Downregulated	−1.65014	0.02515
MIMAT0000787	rno-miR-18a-5p	Downregulated	−1.62661	0.00404
MIMAT0004742	rno-miR-296-3p	Downregulated	−1.60593	0.01964
MIMAT0005284	rno-miR-874-3p	Downregulated	−1.54335	0.00299
MIMAT0000882	rno-miR-211-5p	Downregulated	−1.52523	0.01243
MIMAT0003212	rno-miR-20b-3p	Downregulated	−1.52416	0.00344
MIMAT0005325	rno-miR-598-3p	Downregulated	−1.52143	0.04569
MIMAT0004641	rno-miR-330-5p	Downregulated	−1.51789	0.02862
MIMAT0005302	rno-miR-190b-5p	Downregulated	−1.50745	0.01945

**Table 2:** Primer sequences for miR-122-5p, RNU5G and miR-191-5p.

Accession Number	RNA	Sequence (5′-)
MIMAT0000421	hsa-miR-122-5p	UGGAGUGUGACAAUGGUGUUUG
NR_002852	RNU5G snRNA	AUACUCUGGUUUCUCUUCAGAUCGCAUAAAUCUUUCGCCUUUUACUAAAGAUUUCCGUGGAGAGGAACAACUCUGAGUCUUAACCCAAUUUUUUGAGCCUUGCUCCGACAAGGCUA
MIMAT0000440	hsa-miR-191-5p	CAACGGAAUCCCAAAAGCAGCUG

**Table 3:** Primer sequences for *Igf1r, Casp3, B2m* and *Hprt*.

Accession Number	Gene Name	Symbol	Forward (5′-3′)	Reverse (5′-3′)
NM_052807.2	Insulin-like growth factor 1 receptor	*Igf1r*	GGAATGGGTCGTGGACAGAT	ACAATCAGCAGGATGGCAAC
NM_012922.2	Caspase 3	*Casp3*	GAGCTTGGAACGCGAAGAAAA	AGAGTCCATCGACTTGCTTCC
NM_012512.2	Beta-2-microglobulin	*B2m*	AATTCACACCCACCGAGACC	GCTCCTTCAGAGTGACGTGT
NM_012583.2	Hypoxanthine phosphoribosyltransferase 1	*Hprt1*	GCAGTACAGCCCCAAAATGG	GGTCCTTTTCACCAGCAAGCT

## Data Availability

Not applicable.
